# Recurrent leiomyomatosis peritonealis disseminate: a case report

**DOI:** 10.4314/gmj.v55i2.11

**Published:** 2021-06

**Authors:** Yaw B Mensah, Lawrence Buadi, Afua Abrahams, Andrea A Y Appau, Kwadwo Mensah

**Affiliations:** 1 Department of Radiology, University of Ghana Medical School, Korle Bu, Accra; 2 Department of Obstetrics and Gynaecology, Trauma and Specialist Hospital, Winneba, Ghana; 3 Pathology Department, University of Ghana Medical School, Korle Bu, Accra; 4 Department of Radiology, Korle Bu Teaching Hospital, Korle Bu Accra; 5 Public Health Consultant, P. O Box GP 15533, Kumasi

**Keywords:** Leiomyomatosis peritonealis disseminata, leiomyoma, leiomyosarcoma, recurrent

## Abstract

Leiomyomatosis peritonealis disseminata (LPD), a rare and unusual condition affecting mainly women of reproductive age, causes peritoneal and subperitoneal nodules formed by smooth muscle. Very few cases have been diagnosed since the disease was first described. We present a 42year old female who was managed for infertility and uterine myomata at a Municipal hospital in Ghana. Following a pelvic ultrasound diagnosis of multiple uterine myomata the patient was booked for myomectomy. At surgery to remove her myomata, the patient was found to have several peritoneal nodules some of which were attached to peritoneum, omentum and the surface of bowel loops in addition to a uterine myoma. The disease has since recurred twice after two laparotomies. The diagnosis was made by histopathology of ultrasound-guided biopsy of the nodules, and she has since been on GnRH analogue treatment. LPD simulates peritoneal carcinomatosis; thus, a good history, clinical evaluation, radiological imaging, and histopathologic analysis must be accurately diagnosed. Surgeons' and Radiologists' knowledge of the condition is fundamental to ensuring correct diagnosis and appropriate treatment and to minimising the probability of malignant transformation.

**Funding:**

None declared

## Introduction

Leiomyomatosis peritonealis disseminata (LPD) is a rare and unusual condition associated with multiple vascular peritoneal and subperitoneal nodules formed by smooth muscle cells. Wilson and Peale first described it in 1952. Very few cases have been diagnosed since the disease was described. This is due to its asymptomatic nature hence the possibility of being underdiagnosed.^1^,^2^,^3^,^4^,^5^

LPD is commonly noted in women of reproductive age and rarely in men or postmenopausal women. Patients usually have a history of pregnancy, oral contraceptive use, myomectomy, hysterectomy, uterine leiomyoma, or ovarian tumours.^1^,^2^,^4^,^5^ Clinically, LPD simulates peritoneal carcinomatosis. Accurate diagnosis requires a good history, clinical evaluation and histopathologic analysis. This will ensure appropriate treatment and minimise the probability of malignant transformation.^1^

## Case Report

**Figure 1:**
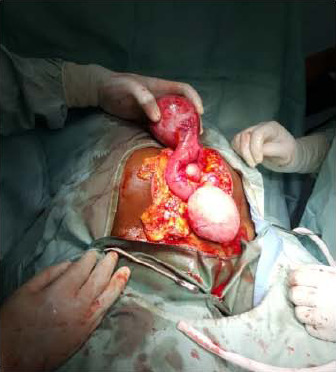
Leiomyomata attached to bowel and omentum during surgery

We present a 42-year-old married nulligravida managed for infertility and abdominopelvic mass in 2016 at the Trauma and Specialist Hospital, Winneba, Ghana. A pelvic ultrasound scan performed diagnosed multiple uterine leiomyomata. She was thus scheduled for myomectomy after the routine laboratory tests were done. At surgery in June 2016, the uterus was about 12 weeks in size with a solitary leiomyoma as well as several nodules (similar to leiomyomata on gross examination) attached to the peritoneum omentum and the surface of bowel loops were noted (Figure 1). The uterine myoma, together with as many of the peritoneal nodules as possible, were removed. She recovered and was discharged.

The patient had previously undergone endometrial polypectomy for abnormal uterine bleeding and anaemia secondary to prolapsed uterine leiomyoma in 2013 at the Korle Bu Teaching Hospital, Accra, Ghana. She continued with her infertility treatment. About eighteen months later, she presented with abdominal distension, which was again diagnosed on abdominopelvic ultrasound due to multiple uterine leiomyomata. A second laparotomy was done in January 2018, which revealed multiple peritoneal nodules, with some in the Pouch of Douglas and on the broad ligaments bilaterally. However, the uterus, which was almost the same size as previously, was fixed due to previous surgery; thus, it was difficult to free it completely to assess its size fully. The nodules were again removed.

**Figure 2:**
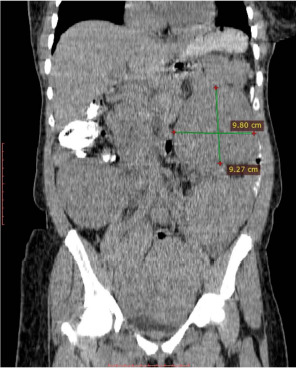
Coronal reformatted abdominal CT scan Image showing multiple isodense round and oval-shaped lesions in the peritoneal cavity before GNRH therapy

About a year later, the abdominal distension and discomfort recurred. The patient's abdomen felt firm and nodular. She had not lost weight. This time the patient was sent for an abdominopelvic computed tomography (CT) scan, which revealed numerous ovoid isodense enhancing lesions distributed diffusely in the peritoneal cavity (Figure 2).

**Figure 3:**
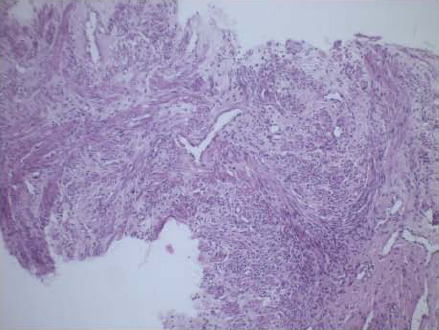
LP (low power) view showing interlacing fascicles of smooth muscle bundles separated by well vascularized connective tissue

**Figure 4:**
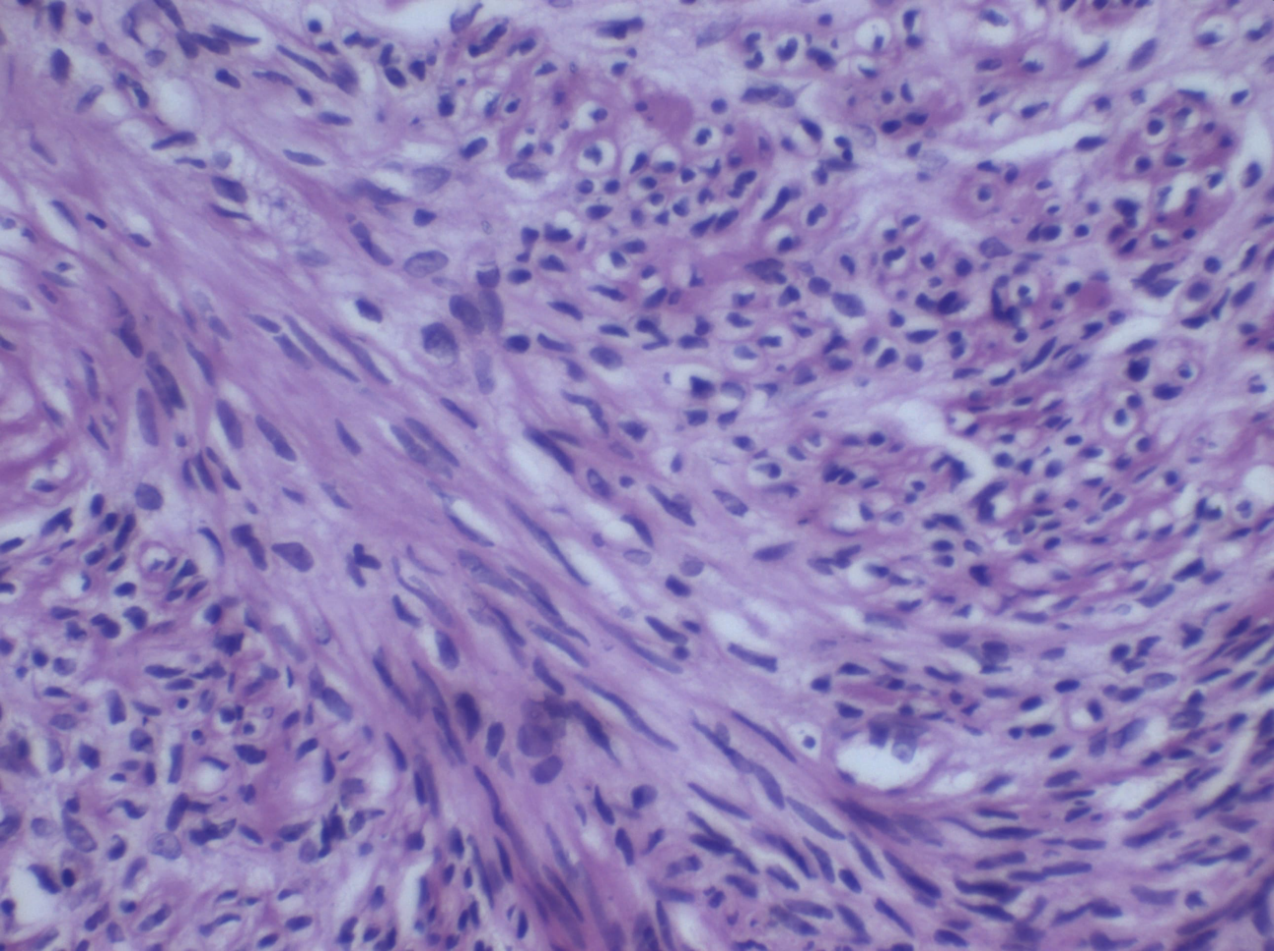
HP (high power) view showing spindle cells with elongated nuclei and eosinophilic or occasional fibrillary cytoplasm and distinct cell membranes

A provisional diagnosis of LPD was made based on the patient's history. There was no family history of this condition. She had a follow-up ultrasound-guided biopsy of the lesions, which showed interlacing fascicles of smooth muscles separated by vascularised connective tissue with no evidence of necrosis or mitosis confirming the diagnosis (Figures 3 and 4).

**Figure 5:**
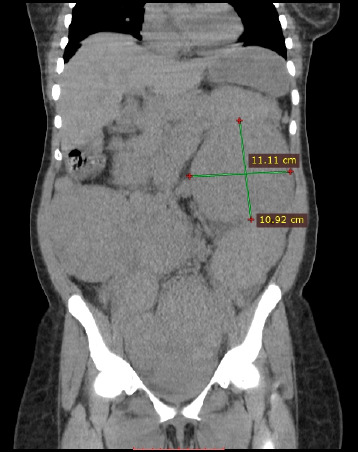
Coronal reformatted abdominal CT scan Images showing multiple isodense round and oval-shaped lesions in the peritoneal cavity after GNRH therapy

She was managed with 3.6mg goserelin acetate a Gonadotropin-Releasing Hormone Analogues (GNRH) per month for three months. She is currently being followed up. A repeat CT scan four months after completing treatment showed an increase in the number and size of the lesions (Figure 5).

## Discussion

This disease was designated as LPD by Taubert et al in 1965.^1^,^5^,^6^ Till date, very few cases have been diagnosed because the disease is asymptomatic and possibly being underdiagnosed.^1^,^4^ Patients are often diagnosed as an incidental finding during a caesarean section or laparotomy for some other indication. She was initially diagnosed with multiple uterine leiomyomata but was noted to have multiple nodules not linked to the uterus during myomectomy.^1^,^4^,^7^

Like the index patient, most of the reported cases of LPD are between 20 years and 55 years. Some LPD patients also have other conditions like leiomyosarcoma, liver leiomyoma, steroid hormone-secreting ovarian tumours and endometriosis;^1^,^4^,^7^,^8^,^9^ she had LPD and leiomyoma. The disease is believed to occur sporadically, and only one family cluster with inherited autosomal dominant inheritance has been reported. The patient denied a family history of LPD, thus supporting the sporadic occurrence theory.^1^,^8^

Though often asymptomatic, some patients may manifest nonspecific symptoms like abdominal pain and discomfort, nausea, vomiting, rectal bleeding, vaginal bleeding, abdominal distention, abdominal masses and intestinal obstruction.^1^ Similarly, she also complained about abdominal distension with discomfort and prolonged bleeding per vaginum.

The aetiology and pathophysiology of LPD is not well understood. Aetiological factors documented are hormonal, subperitoneal mesenchymal stem cell metaplasia, genetic and iatrogenic.^1^,^6^

Lesions are also thought to develop from an unusual and selective sensitivity of mesothelial, submesothelial, multipotential mesenchymal stem cells to hormonal stimulation leading to metaplasia. This phenomenon is believed to be potentiated by the presence of oestrogen receptors (ER) and progesterone receptors (PR) on lesions. The hormonal influence is deduced from pregnancy, prolonged oral contraceptives use, and occasionally, ovarian tumours. In men and postmenopausal women, the condition is attributed partly to the increased responsiveness of tumour cells to normal hormone levels.^1^,^3^,^5^,^6^,^8^,^10^,^11^ Myomectomy and hysterectomy, which fall in the iatrogenic category, are believed to produce leiomyoma fragments that disseminate and implant on the peritoneum and later grow into LPD nodules.^1^,^5^ The history of the index patient make her condition lean more towards this aetiological model than the others aforementioned. This is supported by the fact that she had not received any hormonal therapy before both surgeries.

The LPD nodules are often noted on the mesentery, omentum, peritoneum, Douglas' pouch, the serosal surface of the small and large intestine and rarely involve the entire muscular layer of the colon.^6^ Consistent with what has been documented, the patient also had nodules attached to the peritoneum, omentum, and the bowel's surface.

Pre-operatively, patients are often identified incidentally during imaging for other conditions or because of some of the symptoms above. Imaging modalities like Magnetic resonance Imaging (MRI), Computed Tomography(CT) scan and ultrasound scan can generally detect the lesions of LPD, which tend to have the same features as uterine leiomyoma (Figures 2 and 3).^1^,^2^ However, it is not always easy to distinguish LPD lesions from malignant lesions like leiomyosarcoma and peritoneal disseminated metastasis on imaging, requiring direct sampling with image-guided biopsies for histological diagnosis.^1^,^6^,^12^ As has been documented, the sonographers who initially performed the ultrasound examinations misdiagnosed the nodules as myomata. The surgical findings are in agreement with the reported difficulty in distinguishing LPD from leiomyomata. The abdominopelvic CT scan diagnosed the condition, but it still had to be confirmed histologically, keeping with what has been documented in the literature.

Unlike the malignant lesions, LPD is histologically made of spindle-shaped smooth muscle cells in interdigitating or whorled arrangement with or without mitotic figures. The index patient had a similar pattern, which lacked the nuclear polymorphism, hyperchromasia, tumour cell necrosis and cellular atypia typically noted in malignant lesions.^1^,^8^

LPD must also be distinguished from other benign tumours like benign metastasizing leiomyoma (BML), fbromatosis (desmoid tumour), and gastrointestinal stromal tumour (GIST), but this is sometimes difficult. Immunohistochemistry can help with this differentiation in most cases. LPD nodules tend to show positivity for desmin, actin, caldesmon, Ki-67, vimentin, oestrogen receptor (ER), progesterone receptor (PR), and negativity ckit.^1^,^8^,^12^ Not all the nodules express both the ER and PR simultaneously. Some express only one, while others do not express any of them. Luteinizing hormone receptor has also been detected in LPD cells from a postmenopausal woman who had received tamoxifen therapy for 2 years before the onset of LPD. GIST has CD117-positive cells, thus CD117 should be included in the immunohistochemistry panel for assessing LPD.^6^,^8^,^10^,^12^ Though the patient's imaging and histological findings were consistent with LPD, it would have been ideal to have confirmed the findings with immunohistochemistry. However, financial constraint did not allow that.

There is a possibility of malignant transformation of LPD, though rare. It is believed that the transformation period varies from several months to several years, though no study has been done to confirm this fully. Jain et al. have reported on a patient whose lesions have remained benign after 16 years of follow up. 4,7,10 This patient's biopsy was done 5 years after the initial encounter and the lesions still showed benign features.

There have not been any written guidelines on how LPD is managed. Treatment is often based on the patient's age, comorbidities and the severity of her symptoms. A conservative therapeutic approach for women with reproductive desire, which is based on withdrawal of hormonal stimulus (chemical castration, stopping contraceptive pills), is preferred.^1^,^4^,^6^ GNRH agonists, which have also been used for conservative treatment of LPD^3^ was this patient. A repeat CT scan is done four months after the treatment (Figure 2) showed an increase in size and number of the lesions signifying treatment failure.

Aggressive surgical treatment is recommended in cases of high risk for malignant degeneration. Often these cases do not have a history of exposure to oestrogens or leiomyoma and have negative PR or ER nodules. One such aggressive treatment is bilateral salpingo-oophorectomy which is believed to provide a cure in patients with symptomatic LPD with worrisome gross or histopathological features.^1^,^3^,^8^

## Conclusion

In conclusion, LPD is a rare benign condition which mimics peritoneal carcinomatosis and other malignancies. Accurate diagnosis requires a good history, clinical evaluation, radiological imaging, preoperative image guided-biopsy and sometimes post-operative histopathologic analysis. Surgeons' and Radiologists' knowledge of the condition is fundamental to ensuring correct diagnosis and appropriate treatment and to minimising the probability of malignant transformation.
